# Maternal Filaggrin Mutations Increase the Risk of Atopic Dermatitis in Children: An Effect Independent of Mutation Inheritance

**DOI:** 10.1371/journal.pgen.1005076

**Published:** 2015-03-10

**Authors:** Jorge Esparza-Gordillo, Anja Matanovic, Ingo Marenholz, Anja Bauerfeind, Klaus Rohde, Katja Nemat, Min-Ae Lee-Kirsch, Magnus Nordenskjöld, Marten C. G. Winge, Thomas Keil, Renate Krüger, Susanne Lau, Kirsten Beyer, Birgit Kalb, Bodo Niggemann, Norbert Hübner, Heather J. Cordell, Maria Bradley, Young-Ae Lee

**Affiliations:** 1 Max-Delbrück-Centrum (MDC) for Molecular Medicine, Berlin, Germany; 2 Clinic for Pediatric Allergy, Experimental and Clinical Research Center, Charité Universitätsmedizin Berlin, Berlin, Germany; 3 Klinik fur Kinder- und Jugendmedizin, Technical University Dresden, Dresden, Germany; 4 Department of Molecular Medicine and Surgery, Karolinska Institutet, Stockholm, Sweden; 5 Institute for Social Medicine, Epidemiology and Health Economics, Charité Universitätsmedizin Berlin, Berlin, Germany; 6 Pediatric Pneumology and Immunology, Charité Universitätsmedizin Berlin, Berlin, Germany; 7 Institute of Genetic Medicine, Newcastle University, Newcastle upon Tyne, United Kingdom; 8 Dermatology Unit, Department of Medicine, Solna Karolinska University Hospital, Stockholm, Solna, Sweden; Yale School of Medicine, UNITED STATES

## Abstract

Epidemiological studies suggest that allergy risk is preferentially transmitted through mothers. This can be due to genomic imprinting, where the phenotype effect of an allele depends on its parental origin, or due to maternal effects reflecting the maternal genome's influence on the child during prenatal development. Loss-of-function mutations in the filaggrin gene (*FLG*) cause skin barrier deficiency and strongly predispose to atopic dermatitis (AD). We investigated the 4 most prevalent European *FLG* mutations (c.2282del4, p.R501X, p.R2447X, and p.S3247X) in two samples including 759 and 450 AD families. We used the multinomial and maximum-likelihood approach implemented in the PREMIM/EMIM tool to model parent-of-origin effects. Beyond the known role of FLG inheritance in AD (R1_meta-analysis_ = 2.4, P = 1.0 x 10^−36^), we observed a strong maternal *FLG* genotype effect that was consistent in both independent family sets and for all 4 mutations analysed. Overall, children of *FLG*-carrier mothers had a 1.5-fold increased AD risk (S1 = 1.50, P_meta-analysis_ = 8.4 x 10^−8^). Our data point to two independent and additive effects of *FLG* mutations: i) carrying a mutation and ii) having a mutation carrier mother. The maternal genotype effect was independent of mutation inheritance and can be seen as a non-genetic transmission of a genetic effect. The *FLG* maternal effect was observed only when mothers had allergic sensitization (elevated allergen-specific IgE antibody plasma levels), suggesting that *FLG* mutation-induced systemic immune responses in the mother may influence AD risk in the child. Notably, the maternal effect reported here was stronger than most common genetic risk factors for AD recently identified through genome-wide association studies (GWAS). Our study highlights the power of family-based studies in the identification of new etiological mechanisms and reveals, for the first time, a direct influence of the maternal genotype on the offspring’s susceptibility to a common human disease.

## Author Summary

Most human diseases are caused by a combination of multiple environmental and genetic influences. The widely used case/control approach aims to identify disease risk genes by comparing the genetic constitution of affected and healthy individuals. Although successful, this approach ignores additional mechanisms influencing disease risk. Here, we studied mutations in the filaggrin gene (*FLG*), which are strong risk factors for atopic dermatitis (AD) and allergies, in a large number of families with AD. We found that *FLG* mutations in the mother, not the father, increased the AD risk of the children, even if the child did not inherit the mutation. Thus, our study revealed, for the first time, a direct influence of a maternal mutation on the child’s risk for a common disease. The maternal *FLG* effect was only found when the mothers were allergic, and was absent in families of non-allergic mothers. This finding suggests that *FLG*-induced changes in the maternal immune response shape the child’s immune system during pregnancy and increase the child’s risk for AD. Our study indicates that maternal *FLG* mutations act as strong environmental risk factors for the child and highlights the potential of family-based studies in uncovering novel disease mechanisms in medical genetics.

## Introduction

Atopic dermatitis (AD, eczema) is a chronic inflammatory skin disease with 10–20% prevalence in industrialized countries. The etiology of AD is complex, with multiple genetic and environmental factors influencing disease risk. Genome-wide association studies (GWAS) have successfully identified common genetic variants predisposing to AD, but the effect of these risk loci is small and altogether only account for a fraction of the disease heritability.

The filaggrin gene (*FLG*) encodes a structural protein playing a critical role in the terminal differentiation of the epidermis and in skin barrier function [1]. Loss-of-function mutations in *FLG* were identified as the cause of ichthyosis vulgaris, a common Mendelian trait characterized by dry, scaly skin and frequent AD [2]. Subsequent studies revealed that *FLG* mutations also strongly predispose to AD [3,4]. This observation has been widely replicated, rendering *FLG* the strongest and best characterized AD risk locus to date [1]. Overall, evidence from human and animal studies demonstrated that filaggrin deficiency results in altered skin structure, impaired barrier function and enhanced antigen penetration through the skin, leading to the production of allergen-specific IgE antibodies (specific sensitization) and AD [5–7].

Epidemiological studies on allergic diseases have shown that maternal allergy is a stronger risk factor for the child than paternal allergy [8,9], although some conflicting results have been reported for AD [10,11]. The molecular basis of this preferential maternal transmission of allergy risk is currently unknown but it can potentially occur through two different biological mechanisms, genomic imprinting or direct maternal genotype effects. In genomic imprinting, either the maternally or the paternally inherited allele is expressed while the alternate allele is silenced. Thus, the effect of an allele depends on its parental origin resulting in phenotypic differences between reciprocal heterozygotes (parent-of-origin effects) [12,13]. Recent studies have demonstrated that parent-of-origin effects in complex diseases can be due to genetic variation in imprinted genes [12,13]. Alternatively, maternal genotype effects occur when the maternal genotype directly influences the child’s phenotype. This effect is independent of the child’s own genotype and occurs through the maternally provided environment during prenatal development. Maternal genotype effects can lead to phenotypic differences between reciprocal heterozygotes and are thus considered parent-of-origin effects [13,14].

We hypothesized that loss-of-function mutations in *FLG* may show parent-of-origin effects. Analysis of 2 large family-based cohorts strongly supports that maternal *FLG* mutations directly increase AD risk in the children.

## Results

### Allelic heterogeneity and population-specific mutations in *FLG*

To systematically identify loss-of-function variants at the *FLG* locus, we used data of the Exome Aggregation Consortium (ExAC [15]), which includes whole exome sequencing results of 61,486 individuals. Filtering by frameshift or non-sense mutations revealed 254 loss-of-function mutations in the gene (S1 Table). The majority of *FLG* mutations were very rare, 227 of 254 *FLG* mutations (89,4%) had an allele frequency (AF) < 0.0001. Further analysis revealed the presence of population-specific mutations. For example, the p.L4022X mutation was common in East Asia (AF = 0.02) but absent from all other populations studied. This data confirms and extends previous reports of allelic heterogeneity and population-specific mutations in *FLG* [1].

In the European (non-Finnish) population ExAC reported 146 loss-of-function mutations with a combined AF of 0.052. Of these, the 4 most prevalent mutations, accounting for 86% of all mutant alleles in this population, were selected for genotyping in the present study: p.761fsX35 (c.2282_2285delCAGT; rs558269137, referred to as c.2282del4), p.R501X (c.1501C>T; rs61816761), p.R2447X (c.7339C>T; rs138726443), and p.S3247X (c.9740C>A; rs150597413).

### *FLG* mutations are strong risk factors for AD

The 4 selected mutations were genotyped in 759 complete nuclear families from Central Europe recruited through one or more children with AD (methods and Table 1). The allele frequencies in the founders were in good agreement with previous studies (0.059, 0.034, 0.01 and 0.002 for c.2282del4, p.R501X, p.R2447X and p.S3247X, respectively) [16,17].

**Table 1:** Study populations.

		Central European	Northern European
Families	Total number of families	759	450
	Number of complete nuclear families	759	325
	- families with 1 affected child (Trio)	323	35
	- families with 2 affected siblings (ASP)	399	235
	- families with > 2 affected siblings	37	55
	Number of incomplete families	0	125
	Total number of affected children	1209	680
Unrelated individuals	Number of cases	1147	0
	Number of population-based controls^a^	3339	1854

^a^ All population-based controls were included in the study irrespective of AD status. ASP refers to affected sib pairs.

As previously reported, *FLG* mutations showed a strong over-transmission from heterozygote parents to AD-affected children in a Transmission Disequilibrium Test (TDT; Table 2 [16,17]). We observed no linkage disequilibrium among *FLG* mutations, since each mutation was on a different haplotype and 2 different mutations never occurred together in the same haplotype (S2 Table). Since previous studies reported that these 4 loss-of-function *FLG* mutations have the same effect on AD risk, we decided to merge all variants into a combined genotype [4]. This enabled us to work with one common instead of 4 low-frequency variants. Unless stated otherwise, the results presented below refer to the combined genotype.

**Table 2:** Results of the transmission disequilibrium test.

			Central European				Northern European			
Mutation	A1^a^	A2^b^	Freq^c^	T / U^d^	OR^e^	P^f^	Freq^c^	T / U^d^	OR^e^	P^f^
c.2282del4	del	Wild type	0.059	189 / 91	2.08	< 10^−6^	0.047	83 / 40	2.07	7.26 x 10^−5^
p.R501X	T	C	0.034	97 / 47	2.06	5.70 x 10^−5^	0.012	27 / 13	2.08	0.058
p.R2447X	T	C	0.010	29 / 17	1.71	0.27	0.014	8 / 22	0.36	0.01
p.S3247X	A	C	0.002	6 / 1	6	0.31	0.004	8 / 6	1.33	1
Combined	Mutation	Wild type	0.105	316 / 150	2.11	< 10^−6^	0.085	125 / 78	1.60	0.001

^a^ A1 is the mutant allele.

^b^ A2 is the wild type allele.

^c^ Allele frequency of A1 in the family founders.

^d^ Number of transmitted (T) and un-transmitted (U) A1 alleles.

^e^ Odds ratio calculated as the ratio of transmitted versus un-transmitted alleles.

^f^ Empirical p value calculated by flipping transmitted/untransmitted status in order to account for multiple affected siblings in each family.

### Direct maternal genotype effect of *FLG* mutations

Testing for parent-of-origin effects was performed with the PREMIM/EMIM software. This tool uses a multinomial model-based maximum-likelihood approach for flexible modelling of parent-of-origin effects (see methods) [18,19]. In order to increase power, we included *FLG* genotypes of 1147 unrelated AD cases and 3339 population-based controls. This set of unrelated individuals does not provide information on parent-of-origin effects, but increases the power to detect them by improving the estimation of genotype frequencies in the general population (see methods) [18,19].

We performed a step-by-step analysis starting with a basic genetic model and successively including additional risk parameters modelling parent-of-origin effects. The basic scenario ignored the available parental genotypes and tested the effect of the child´s genotype on his own phenotype. As expected, we observed large effects with relative risks of 3.1 for heterozygous (R1 parameter) and 10.5 for homozygous *FLG* mutation carriers (R2 parameter) (Child Genotype or CG model; R1 = 3.1, R2 = 10.5; *P*_CG_ = 5.9 x 10^−74^; Table 3).

**Table 3:** Parent-of-origin analysis of the combined *FLG* mutations.

**Children Genotype model (CG)**						
Study	R1 (CI)	R2 (CI)	S1 (CI)	Im (CI)	*P*_null_ ^a^	-
Central Europe	3.10 (2.68–3.59)	10.5 (7.20–15.36)	-	-	5.9 x 10^−74^	-
Northern Europe	2.44 (1.95–3.06)	7.37 (3.37–15.81)	-	-	9.23 x 10^−17^	-
Meta-analysis *P*_meta_ ^c^	2.89 (2.56–3.27) 2.8 x 10^−65^	9.80 (6.97–13.77)2.1 x 10^−39^	-	-	-	-
*P*_het_ ^d^	0.08	0.41				
**Maternal Child Genotype model (MCG)**						
Study	R1 (CI)	R2 (CI)	S1 (CI)	Im (CI)	*P*_null_ ^a^	*P*_MCG vs CG_ ^b^
Central Europe	2.57 (2.18–3.04)	7.97 (5.36–11.87)	1.55 (1.29–1.87)	-	2.7 x 10^−77^	5.0 x 10^−6^
Northern Europe	2.13 (1.67–2.72)	5.89 (2.67–12.97)	1.42 (1.11–1.82)	-	1.42 x 10^−17^	0.005
Meta-analysis *P*_meta_ ^c^	2.43 (2.11–2.78) 1.0 x 10^−36^	7.50 (5.26–10.70) 1.2 x 10^−28^	1.50 (1.29–1.74) 8.4 x 10^−8^	-	-	-
*P*_het_ ^d^	0.21	0.50	0.58			
**Imprinting model (Im)**						
Study	R1 (CI)	R2 (CI)	S1 (CI)	Im (CI)	*P*_null_ ^a^	*P*_Im vs CG_ ^b^
Central Europe	2.70 (2.19–3.31)	8.06 (5.09–12.78)	-	1.30 (1.00–1.69)	1.2 x 10^−73^	0.047
Northern Europe	2.19 (1.63–2.95)	5.97 (2.56–13.92)	-	1.23 (0.86–1.75)	3.22 x 10^−16^	0.25
Meta-analysis *P*_meta_ ^c^	2.52 (2.13–2.99) 1.4 x 10^−26^	7.53 (5.02–11.28) 1.4 x 10^−22^	-	1.28 (1.03–1.57) 0.02	-	-
*P*_het_ ^d^	0.26	0.54	0.80			

^a^ *P* value for the comparison of each model versus the null model with no effects.

^b^ *P* value for the comparison of each model versus the Child Genotype model.

^c^ *P* value for the meta-analysis of each estimated parameter (see methods). CI indicates 95% confidence interval.

^d^ P value for a test of heterogeneity. All results correspond to the combined *FLG* mutations.

Next maternal genotype effects were modelled by including an additional parameter, S1, to estimate the relative AD risk of children whose mother carried a *FLG* mutation. Children of *FLG* mutation-carrier mothers had a striking 1.55 fold increase in AD risk independently of their own genotype (Mother-Child Genotype model or MCG; R1 = 2.57, R2 = 7.97, S1 = 1.55; *P*_*M*CG_ = 2.7 x 10^−77^; Table 3). A comparison of the Child Genotype and the Mother-Child Genotype models by a likelihood-ratio test, strongly supported the existence of a maternal genotype effect (*P*_*MCG vs CG*_ = 5.0 x 10^−6^). In addition, we observed no evidence of interaction between the child and maternal genotypes, indicating that carrying a mutation and having a mutation carrier mother are independent risk factors with additive effect on disease risk (S3 Table and methods). Thus, children with both risk factors, i.e. carrying a *FLG* mutation and having a mutation carrier mother, have a nearly 4-fold increased disease risk (R1 x S1 = 3.6).

Importantly, both genomic imprinting and maternal genotype effects can lead to similar patterns of parent-of-origin effects and specific tests need to performed to distinguish them [13,14]. Finally, we tested an imprinting model by including the imprinting parameter, Im, which represents the relative risk of the child when inheriting a mutant allele from the mother as opposed to the father. A comparison with the Child Genotype model provided marginal support for the presence of imprinting (*P*_*Im vs CG*_ = 0.047; Table 3). In order to test which parent-of-origin scenario better fits our data we performed comparisons versus a full model containing all risk parameter (R1, R2, S1 and Im). Interestingly, adding the maternal genotype parameter S1 to a model already containing Im resulted in a significantly better model (Table 4; p = 3.2 x 10^−6^). On the contrary, adding Im to a model already containing S1 provided only a marginal improvement (p = 0.03). These results favour the existence of a direct maternal genotype effect of *FLG*.

**Table 4:** Comparison of the MCG and Imprinting models with the full model.

	Parameters included				Central European study				Northern European study			
Model	R1	R2	Im	S1	lnlik	*P*_null_ ^a^	*P*_CG_ ^b^	*P*_full_ ^c^	lnlik	*P*_null_ ^a^	*P*_CG_ ^b^	*P*_full_ ^c^
Null	-	-	-	-	−3378.5				−1376.3			
CG	+	+	-	-	−3209.89	5.9 x 10^–74^			−1343.5	x 10^−17^		
MG	+	+	-	+	−3199.46	2.7 x 10^−77^	5.0 x 10^−06^	2.9 x 10^−02^	−1339.8	1.4 x 10^−17^	0.005	0.162
Im	+	+	+	-	−3207.92	1.2 x 10^−73^	4.7 x 10^−02^	3.2 x 10^−06^	−1342.3	3.3 x 10^−16^	0.25	0.004
Full	+	+	+	+	−3197.07	2.9 x 10^−77^	2.7 x 10^−06^		−1339.5	3.1 x 10^−17^	0.008	

The “+” and “-” symbols indicate whether a given risk parameter was included or excluded in the corresponding model. lnlik is the maximized ln likelihood for each model.

^a^ P value for the comparison of each model versus the null model with no effects.

^b^ P value for the comparison of each model versus the child genotype model including R1 and R2.

^c^ P value for the comparison with the full model including all risk parameters (R1, R2, S1, S2, and Im).

### Replication in an independent sample and meta-analysis

We aimed to replicate our findings by examining the same 4 *FLG* mutations in an independent Northern European population including 450 AD families and 1854 population-based control individuals (methods and Table 1) [17,20–22]. Step-by-step analysis with PREMIM/EMIM again supported a maternal genotype effect. The genotypes of both children and mothers had an independent effect on AD risk, and children of *FLG*-carrier mothers showed a 1.4 fold increased risk (R1 = 2.13, R2 = 5.89, S1 = 1.42; *P*_*M*CG_ = 1.4 x 10^−17^; *P*_*MCG vs CG*_ = 0.005; Table 3 and S3 Table). Importantly, the results obtained were consistent in both populations studied providing strong support to the existence of maternal genotype effects on F*LG*.

A meta-analysis was performed using the inverse variance method as implemented in METAL [23], which uses the effects estimates and standard errors from each risk parameter. This revealed a highly significant 1.5 fold increased AD risk in children of *FLG*-carrier mothers (S1_meta-analysis_ = 1.50; *P =* 8.4 x 10^−8^; Table 3).

### *FLG* mutations are more frequent in mothers than in fathers

**Fig 1:**
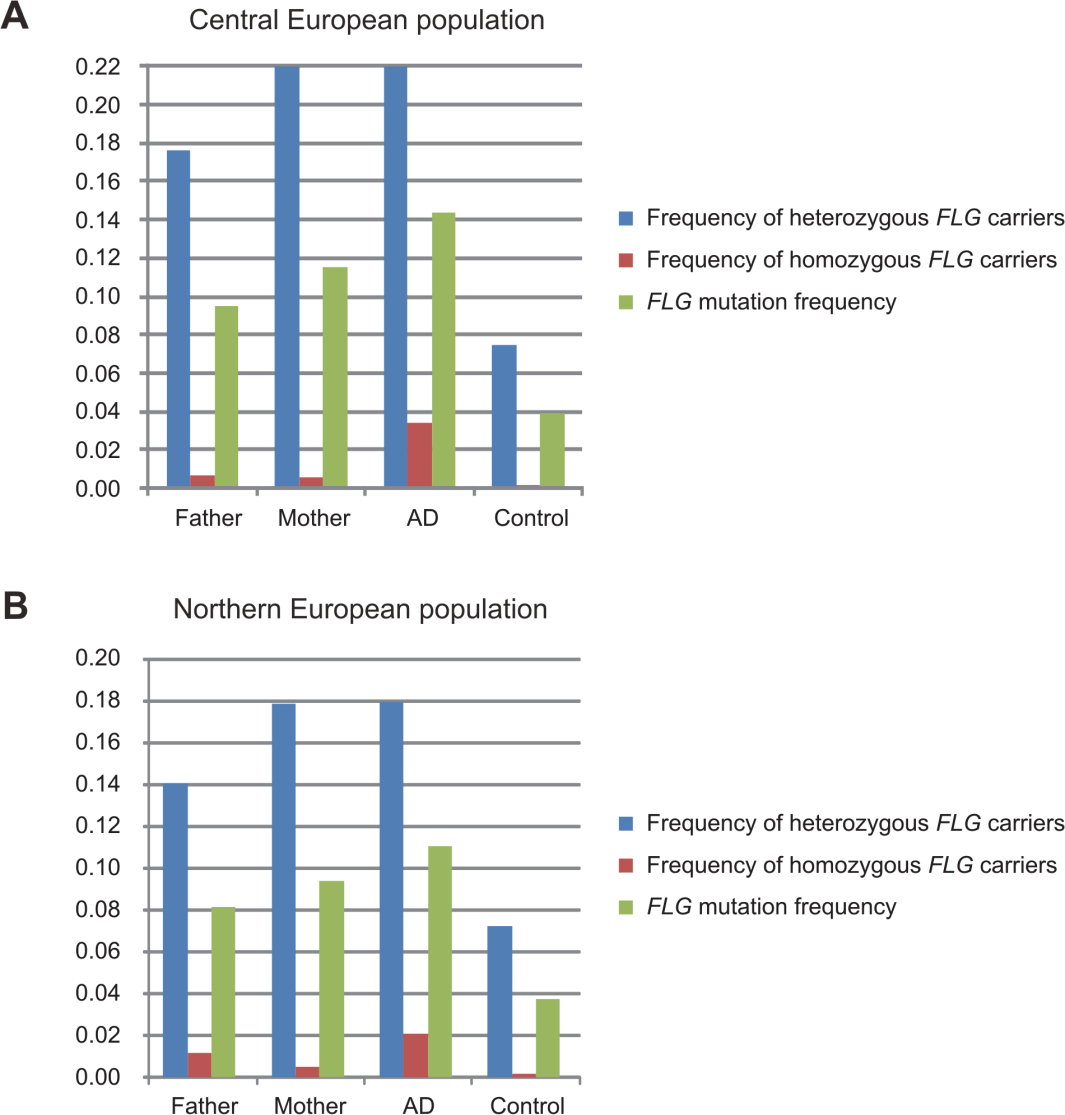
Frequency of FLG mutations in fathers, mothers, individuals with atopic dermatitis and controls. Allele and genotype frequencies of the combined *FLG*-mutations in fathers and mothers were calculated using all available parents. AD refers to the frequency in the AD-affected children including the families and the unrelated AD-cases (available only in the Central European study). Frequency in controls corresponds to population-based individuals with unknown disease status. Results of the Central and Northern European populations are shown in panels A and B, respectively.

Analysis of parental genotypes revealed a higher prevalence of *FLG* mutations in mothers than in fathers in both study populations (Fig. 1). This is consistent with the maternal genotype effect observed. Additionally the frequency of *FLG* mutations in the parental population (mothers and fathers together) was remarkably higher than in population-based controls of unknown phenotype. This is likely due to the recruitment of families with multiple affected children leading to a parental population enriched in strong genetic risk factors.

### Robustness of the *FLG* maternal effect

At this stage we considered the potential weaknesses of our study in order to discard false positives due to methodological issues and to gain further support to the existence of a *FLG* maternal genotype effect.

We analysed the *FLG* c.2282del4, p.R501X and p.R2247X mutations independently (this was not possible for p.S3247X since it was too rare). The maternal effect and the increased frequency of mutations in mothers were found for all 3 mutations in both study populations (S4 Table and S5 Table). This suggests that the maternal effect is not specific to a given variant but a general characteristic of *FLG* loss-of-function mutations.

In the populations studied, mothers typically have a more prominent role than fathers in children’s health care [24]. We hypothesized that preferential ascertainment of AD-affected mothers carrying *FLG* mutations may be the cause of the observed maternal effect. Indeed, we observed a higher frequency of AD-affected mothers than fathers, which could be due to a genuine maternal effect or to ascertainment bias (AD prevalence in Central European mothers = 0.23 and fathers = 0.12; Northern European mothers = 0.35 and fathers = 0.19). In order to avoid this potential bias we repeated our analysis including only families in which both parents had a negative history of AD. Importantly, the maternal genotype effect remained strong and significant in the remaining population (Meta-S1 = 1.38; *P =* 0.003; S6 Table).

We also tested the potential effect of the paternal *FLG* genotype on the children. Since this option is not available in PREMIM/EMIM, we performed the analysis after exchanging the paternal and maternal genotypes on our genotype files. This analysis revealed no significant effect of the paternal *FLG* status (S7 Table).

A large proportion of the families included in the present study (60%) were recruited through an affected sib pair. Aiming to maximize power, all previous analyses were performed considering all affected siblings as independent individuals which may lead to biased risk parameter estimates. We therefore repeated the analysis including only one affected child per family and found that the magnitude of the maternal effect remained constant in this set of independent trios (S1_meta-analysis_ = 1.45; *P =* 1.1 x 10^−4^; S8 Table).

### Potential influence of maternal immunity

Filaggrin has a major role in cutaneous barrier function [1]. According to publicly available datasets [25–27] *FLG* expression is highest in skin and absent in tissues relevant for mother-child interactions such as uterus, placenta, or mammary gland (S1 Fig. and S2 Fig.). However, recent studies demonstrated that *FLG* mutations result in increased antigen penetration through the skin and the production of allergen-specific antibodies (IgE, specific sensitization) [6,7,28]. Thus, we hypothesized that systemic inflammatory responses in *FLG* carrier mothers may influence AD risk in children via feto-maternal immune crosstalk.

All available maternal plasma samples from the Central European study were therefore tested for the presence of allergen-specific IgE, which is a well-characterized biochemical marker of allergy [29]. We performed a stratified analysis in 253 families with and 311 families without maternal specific sensitization. In the families with maternal specific sensitization (+Mat_sens) we observed a strong maternal genotype (S1 = 1.63; *P*_*MCG vs CG*_ = 0.005) and a weak child genotype effect (R1 = 1.37; ; P_null_ = 0.03; Table 5). This was consistent with a marginal over-transmission of *FLG* mutations from parents to affected offspring in a transmission disequilibrium test (TDT, transmitted (T): untransmitted (U) = 97:65, *P =* 0.04; Table 6). In contrast, the opposite pattern was observed in the group of families with non-allergic mothers (−Mat_sens). Here, the maternal genotype effect was not significant (S1 = 1.18; *P*_*MCG vs CG*_ = 0.3) while the child genotype effect was strong (R1 = 2.3; P_null_ = 9.9 x 10^−11^). This observation was confirmed by a striking over-transmission of *FLG* mutations in the TDT (T:U = 169:68, *P =* 5.3 x 10^−8^). In concordance with the different rates of mutation transmission observed in both groups, the frequency of *FLG* mutations was significantly higher in AD-children of non-allergic mothers (mutation frequency 0.16 and 0.11 in affected children of −Mat_sens and +Mat_sens families, respectively; OR = 1.48; *P =* 0.005; Table 6).

**Table 5:** Parent-of-origin-analysis analysis stratified by maternal specific sensitization.

**Children Genotype model (CG)**						
Group	n^a^	R1 (CI)	R2 (CI)	S1 (CI)	*P*_null_ ^b^	*P*_MCG vs CG_ ^c^
+Mat_sens	253	1.37 (0.97–1.94)	2.98 (1.19–7.45)	-	0.03	-
−Mat_sens	311	2.30 (1.64–3.22)	7.19 (3.77–13.7)	-	9.9 x 10^−11^	-
**Maternal Child Genotype model (MCG)**						
Group	n^a^	R1 (CI)	R2 (CI)	S1 (CI)	*P*_null_ ^b^	*P*_MCG vs CG_ ^c^
+Mat_sens	253	1.37 (0.97–1.93)	2.97 (1.19–7.43)	1.63 (1.15–2.31)	0.002	0.005
−Mat_sens	311	2.30 (1.64–3.22)	7.19 (3.77–13.69)	1.18 (0.86–1.61)	3.2 x10^−10^	0.30

^a^ Number of families.

^b^ *P* value for the comparison with the null model with no effects.

^c^ *P* value for the comparison with the Child Genotype model.

CI indicates 95% confidence interval. All results correspond to the combined *FLG* mutations. Note that all unrelated cases and population-based controls were excluded from this analysis due to missing maternal sensitization data.

**Table 6:** TDT analysis stratified by maternal specific sensitization.

Transmission Disequilibrium Test (TDT)					Allelic test in children ^d^	
Group	n^a^	T / U^b^	OR^c^	*P*	Mut freq	OR (*P*)
+Mat_sens	253	97 / 65	1.49	0.04	0.11	1
−Mat_sens	311	169 / 68	2.49	5.3 x10^−8^	0.16	1.48 (0.005)

^a^ Number of families.

^b^ Number of transmitted (T) and un-transmitted (U) alleles. Allele counts are referred to the combined *FLG* mutations.

^c^ Odds ratio calculated as the ratio of transmitted versus un-transmitted alleles.

^d^ Comparison of allelic frequency between AD-affected children of the −Mat_sens versus the +Mat_sens families.

## Discussion

We report here that maternal loss-of-function mutations in *FLG* directly influence AD risk in the offspring independently of the child’s own genotype. Importantly, this maternal effect was observed consistently for all different *FLG* mutations tested and in 2 independent populations. Given that genomic imprinting and maternal genotype effects can lead to similar patterns of parent-of-origin effects [14], we specifically modelled both scenarios. Although we cannot completely exclude an imprinting effect, our data supports a direct maternal genotype effect of *FLG* mutations. This is consistent with the lack of known imprinted genes in the 1q21.3 genomic region containing *FLG* [25].

It is not obvious how maternal mutations in a skin-barrier gene can influence the child’s phenotype. However, given that filaggrin deficiency promotes specific sensitization, we hypothesized that systemic immune responses may play a role in the *FLG* maternal effect. Our observation that the *FLG* maternal effect is significant only in the group of sensitized mothers supports this hypothesis and indicates that *FLG* mutations in allergic mothers act as a strong environmental risk factor for AD in the child.

The importance of prenatal mother-child interaction in shaping the child’s immune phenotype is highlighted by studies in pregnant mice: while the induction of Th2 immune responses increased susceptibility to allergic asthma in the offspring, a protective effect was observed upon induction of Th1 responses, emphasizing the importance of maternal immune status during pregnancy [30–32]. Supporting evidence in humans arise from epidemiological studies showing that exposure to a microbial-rich farm environment during pregnancy protects children from the development of allergic diseases [33]. It is unknown how this “immunological imprinting” may be transmitted from mother to child, but animal and human studies suggest the induction of epigenetic modifications in relevant immune cells in the offspring [31,34]. Future large population-based studies with parental DNA, data on maternal allergic sensitization and biological material available for epigenetic analysis will be required to further explore this interesting hypothesis.

A recent mouse study found that parent-of-origin effects are widespread and account for an unexpectedly large proportion of complex trait heritability [35]. This is supported by human studies demonstrating that the parental origin of an allele inherited by the offspring can affect disease susceptibility to complex diseases [12,13,36–38]. However, evidence for the existence of maternal genotype effects, which occur without transmission of the risk allele to the offspring, comes mainly from animal studies analysing the effect of maternal gene knockouts in wild-type offspring [39]. Examples of such effects in humans are scarce and refer to rare congenital malformations [40,41]. The present work is, to our knowledge, the first report of a large maternal genotype effect in a common human disease. Notably, the magnitude of the maternal effect (RR = 1.5) was consistent in both data sets and exceeded that reported for most AD genetic risk factors identified to date.

Interestingly, AD is commonly the first manifestation of allergic disease and filaggrin deficiency is a risk factor for the transition from AD to other atopic diseases such as food allergy, hay fever, and asthma [5,16]. Thus, it is tempting to speculate that maternal *FLG* mutations may influence the risk of a much wider range of allergic disorders.

This and other studies provide proof-of-principle that associations originally discovered by case-control analysis can arise as a consequence of parent-of-origin effects, although with an underestimation of the effect size due to inaccurate genetic modelling [12]. Family-based studies re-evaluating previously identified susceptibility loci will enable the identification of parent-of-origin effects and help characterize part of the missing heritability in complex traits.

## Methods

### Ethics statement

The study was carried out in accordance to the approval of the ethics commission of the Charité—Universitätsmedizin Berlin (ref EA2/054/10) and following the guidelines of the declaration of Helsinki. Informed consent was obtained from all probands or their legal guardians.

### Subjects

We investigated samples originating from European family-based and population-based studies. All samples were divided, according to the country of origin, into a Central and a Northern European study population (Table 1).

The GENUFAD study (Genetic Analysis of Nuclear Families with Atopic Dermatitis) recruited complete nuclear families with at least two children affected with early-onset (<2 years of age) and moderate to severe AD as previously described [20]. A doctor’s diagnosis of AD was made according to standard criteria [42]. The GENUFAD study contributed 522 complete German nuclear families to the Central European study and 32 Swedish families to the Northern European data set. A large proportion of these families have been reported in previous studies [16,43].

The MAS (Multicenter Allergy Study) is a previously described population-based birth cohort in which 1314 German infants were followed since 1990 to investigate the epidemiology of allergic diseases [44]. The diagnosis of AD was made as previously described [16]. 112 German MAS trios, consisting of a child with AD and both parents, were included in the Central European study.

The ETAC (Early Treatment of the Atopic Child) is a European study which recruited infants diagnosed with AD in their first year of life into a randomized, double blind, placebo controlled trial on the efficacy of cetirizine in the prevention of asthma [45]. Children with early onset and moderate to severe disease were selected for the present study when parental DNA was available. The ETAC study contributed 21 Swedish trios to the Northern European study. Additionally, 125 ETAC trios from different European countries were included in the Central European study (48 from the Netherlands, 23 from Italy, 20 from the UK, 15 from France, 13 from the Czech Republic, and 6 from Germany).

A previous study from Sweden contributed 397 families to the Northern European study group [17]. This included 272 complete affected sib pair families with AD diagnosed according to the U.K. Working Party’s Diagnostic Criteria [46]. The remaining 125 families were incomplete nuclear families including mother-child or father-child pairs.

In all family-based studies, information regarding the parental history of AD was obtained by a questionnaire at the time of family recruitment. The analysis shown in S6 Table was performed in families in which both parents had a negative history of AD. Families in which one or both parents had a positive or unknown disease history were excluded.

As described below, the analytical methods used allowed the incorporation of unrelated individuals to increase power. Thus, the Central European study also included previously published *FLG* genotypes of 772 unrelated German AD cases and 373 German controls from a previous GWAS [43]. In addition, we genotyped *FLG* mutations in 375 German children with AD diagnosed at a tertiary care center for pediatric allergy at Charité Universitätsmedizin Berlin. Also, previously published *FLG* genotype counts of 2,963 population-based German individuals from the International Study of Asthma and Allergies in Childhood II Study [47] were included. Likewise, in our Northern European study population we included genotypes of the 3 most prevalent *FLG* mutations (c.2282del4, p.R501X and p.R2447X) previously reported in the Swedish population-based BAMSE cohort [22]. The rarest *FLG* mutation (p.S3247X) was not available in the BAMSE dataset.

Data on specific allergic sensitization was available in a large proportion of mothers from the GENUFAD and MAS studies. Plasma levels of specific IgE against grass and birch pollen, ribwort, cat and dog dander, mold (*Cladosporidium herbarum, Alternaria tenuis*), hen’s egg, cow’s milk, fish, peanut, and house dust mite were determined using CAP-RAST-FEIA (Pharmacia). A mother was defined as sensitized if specific IgE ≥0.7 kU/l (CAP2) to at least one allergen was detected.

### Genotyping of *FLG* mutations

Genomic DNA was prepared from whole blood by standard methods. The *FLG* c.2282del4 variant was analyzed with fluorescence-based semiautomated genotyping [16] and the *FLG* p.R501X, p.R2447X and p.S3247X mutations with Taqman allelic discrimination (Applied Biosystems, Foster City, California, USA) as previously described [4]. Genotyping of p.R2447X in the Northern European families was performed using a fluorescent Kaspar assay (KASP-By-Design genotyping assays, LGC group, Teddington, UK). Genotyping with Taqman and Kaspar was performed using a ViiA 7 Real-Time PCR System (Applied Biosystems, Foster City, California, USA). When analyzing the rare p.R2447X and p.S3247X mutations, a sample known to be a mutation carrier was included on each genotyping plate as a positive control.

The *FLG* mutations are named according to the nomenclature recommendations by den Dunnen and Antonarakis [48]. The positions of the mutations in the cDNA refer to the A of the ATG-translation initiation codon of NM_002016.1.

### Statistical analysis

The PREMIM and EMIM tools were used to test for imprinting and maternal genotype effects of the *FLG* mutations [18,19]. First, the PREMIM tool was used to classify each trio according to the number of copies of *FLG* mutations carried by mother, father, and affected child. Incomplete nuclear families, unrelated AD cases, and population-based samples were also included in the analysis to increase the power to detect parent-of-origin effects [18,19]. Since the EMIM analysis is based on the assumption that the genotype frequencies in controls correspond to those in the general population, all population-based controls were included in the analysis irrespectively of disease status. Starting values for allele frequencies of the *FLG* mutations in the study population (including controls) were estimated with PREMIM (–a option).

The trios were analyzed using the EMIM tool, which uses a multinomial modelling approach to estimate genotype relative risk parameters on the basis of observed counts of genotype combinations in case-parent trios. The following parameters influencing the disease risk in the child were modelled with EMIM:

- R1 (R2), the factor by which an individual’s disease risk is multiplied if they carry one (two) risk alleles at a given locus.
- S1 (S2), the factor by which an individual’s disease risk is multiplied if the mother carries one (two) risk alleles at that locus.
- Im (Ip) the factor by which an individual’s disease risk is multiplied if inheriting a risk allele from the mother (or father).
- γ11 (interaction term), the factor by which an individual’s disease risk is multiplied if both mother and child have 1 copy of the risk allele.

Previous data indicated that *FLG* mutations do not fit an additive genetic model, since the risk of AD in the homozygous carriers is too high. Thus, instead of using the default EMIM settings assuming an additive model we choose to independently estimate the R1 and R2 parameters. Modelling of maternal genotype effects was done using the default additive model. All analyses were performed under the “conditional on exchangeable parental genotype” (CEPG) assumption. This assumption should protect from potential biases in parameter estimation due to the inclusion of families recruited through multiple affected individuals, at the cost of reduced power to detect parent-of-origin effects compared to assuming only Hardy-Weinberg equilibrium [18,19]. A step-by-step analysis was performed by including additional risk parameters in the model as indicated in S9 Table.

Maximum likelihood estimates were obtained from each model and a likelihood ratio test was performed to assess the significance among nested models. Note that it is not possible to directly compare the MCG and Im models in a likelihood ratio test, since they are not nested. However, they can be compared indirectly by comparison to the full model (Table 4).

In order to test for genetic interaction between the child and the maternal genotypes we included and interaction term in the model (γ11). This parameter estimates the factor by which an individual’s disease risk is multiplied if both mother and child have one copy of the risk allele. A likelihood ratio test comparing the Maternal Child Genotype (MCG) and the MCG-Interaction model was then performed (see S10 Table).

A meta-analysis of the results from the Central and Northern European populations was performed using METAL [23]. The inverse variance method was used and the corresponding betas and standard errors were obtained from the EMIM summary file. The meta-analysis was performed on the single risk parameters estimates (R1, R2, S1 or Im) from each model. An analysis of heterogeneity was also performed with METAL in order to evaluate if the observed effect sizes were homogeneous across datasets.

In order to increase power in the main analysis we allowed the inclusion of all affected offspring available in each family which were considered as independent trios (-xa option in PREMIM). This may lead to biased results when using large pedigrees but it is unlikely to have a large effect in our study since most families had only 1 or 2 affected children. In order to exclude this potential bias we repeated the analysis including one affected child per family (omitting the −xa option in PREMIM; S8 Table).

The Transmission Disequilibrium Test (TDT) was performed with PLINK [49]. In order to account for multiple affected offspring within families, empirical p-values were calculated with the —tdt —perm option, which flips the allele transmitted from parent to offspring with 50:50 probability. Allelic effects were calculated with PLINK —assoc using the offspring of the +Mat_Sens as controls and those from the −Mat_Sens as cases.

Haplotype frequencies on the central European Study were calculated with FAMHAP [50], which computes maximum-likelihood estimates obtained with the expectation-maximization algorithm.

## Supporting Information

S1 Fig Tissue expression pattern of *FLG* from BioGPS. Data from the BioGPS Portal where mRNA levels were quantified with expression arrays in human tissues. [25,26].
(TIF)

S2 Fig Tissue expression pattern of *FLG* from GTEx. Data from the GTEx Consortium where mRNA was quantified by Next generation Sequencing. [**27**]
(TIF)

S1 Table *FLG* loss-of-function mutations in the ExAC project. Only high quality mutations were considered (filter = pass). ^a^ Human genome build GRCh37.p13 (chromosome 1). ^b^ Alias used in this manuscript. ^c^ Allele frequency in the combined set of 61,486 unrelated individuals. ^d^ Alternative allele count of genotypes in the combined population ^e^ Total number of called genotypes in the combined population. Data was accessed on December 12, 2014.
(XLSB)

S2 Table Lack of linkage disequilibrium among *FLG* mutations. (DOCX)

S3 Table Analysis of interaction between child and maternal *FLG* mutations. (DOCX)

S4 Table Analysis of individual *FLG* mutations. (DOCX)

S5 Table Frequencies of each of the 4 *FLG* mutations analyzed independently. (DOCX)

S6 Table Parent-of-origin analysis after exclusion of families with parental history of AD. (DOCX)

S7 Table Analysis for paternal genotype effects. (DOCX)

S8 Table Parent-of-origin analysis with one single AD-affected child per family. (DOC)

S9 Table Step-by-step analysis with PREMIM/EMIM. (DOCX)

S10 Table Models testing interaction between the child and the maternal genotypes. (DOCX)
