# Supplementary figures and images for: Maternal Filaggrin Mutations Increase the Risk of Atopic Dermatitis in Children: An Effect Independent of Mutation Inheritance
Source: PLoS Genet. 2015 Mar 10;11(3):e1005076. doi: 10.1371/journal.pgen.1005076 (PMC4355615; doi:10.1371/journal.pgen.1005076)

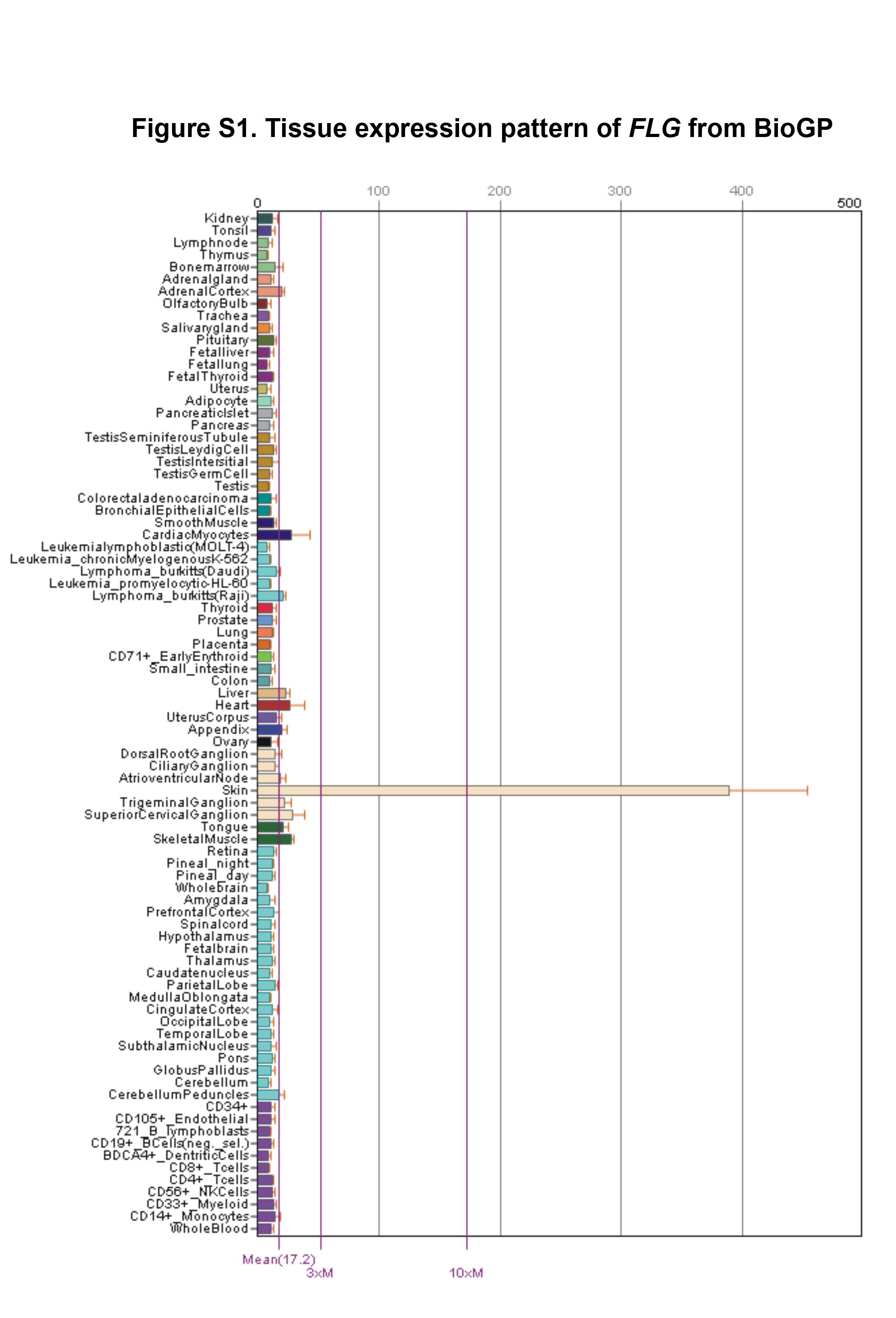

Supplement: S1 Fig — Data from the BioGPS Portal where mRNA levels were quantified with expression arrays in human tissues. [25,26]. (TIF) [file pgen.1005076.s001.tif]

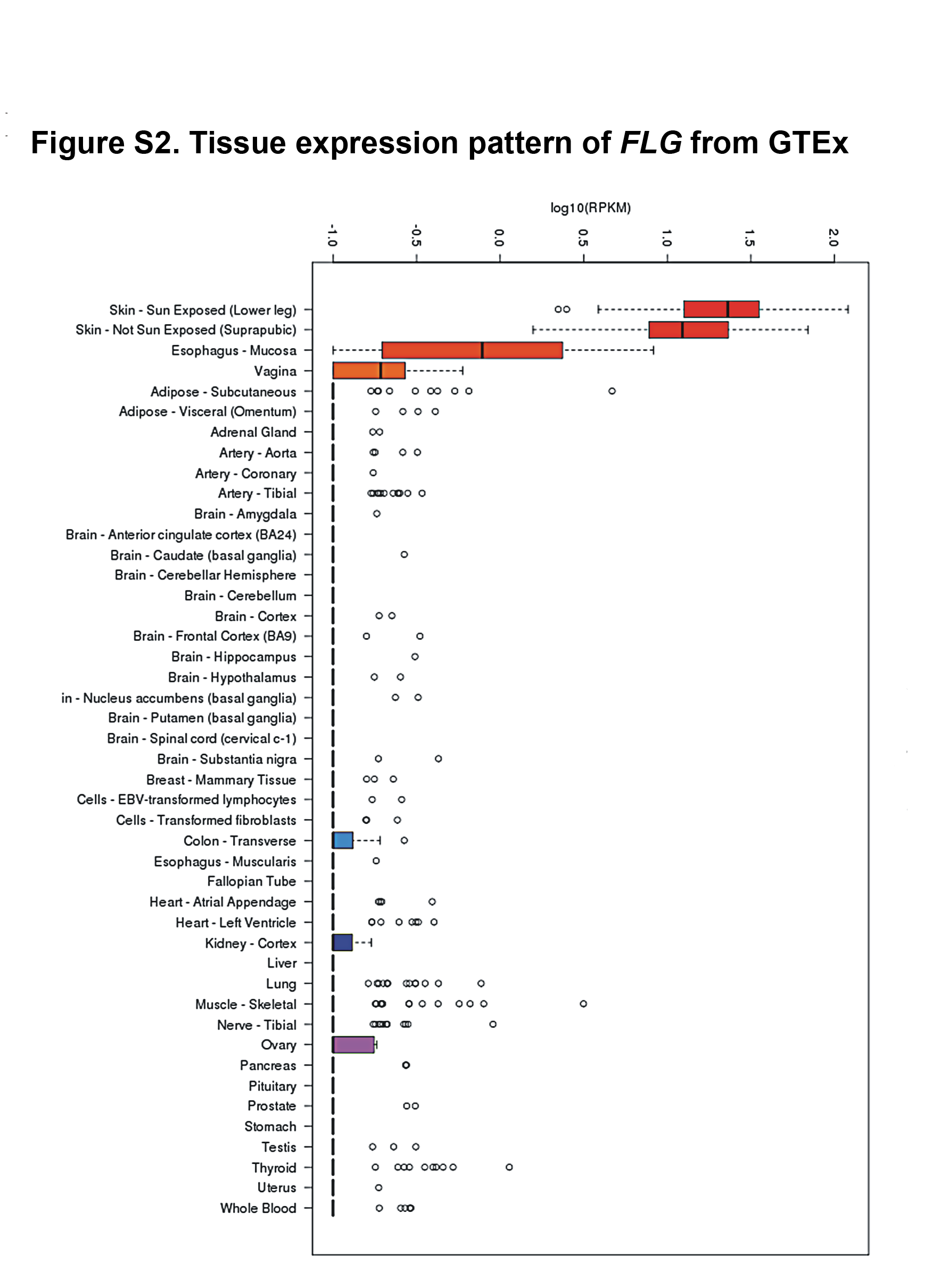

Supplement: S2 Fig — Data from the GTEx Consortium where mRNA was quantified by Next generation Sequencing. [27] (TIF) [file pgen.1005076.s002.tif]
