# Supplementary material for: Maternal Filaggrin Mutations Increase the Risk of Atopic Dermatitis in Children: An Effect Independent of Mutation Inheritance
Source: PLoS Genet. 2015 Mar 10;11(3):e1005076. doi: 10.1371/journal.pgen.1005076 (PMC4355615; doi:10.1371/journal.pgen.1005076)
Supplement: S2 Table — (DOCX) [file pgen.1005076.s004.docx]

**Table S2: Lack of linkage disequilibrium among FLG mutations.**

|  | *FLG* mutation | | | |  |
| --- | --- | --- | --- | --- | --- |
| Haplotype | c.2282del4 | p.R501X | p.R2447X | p.S3247X | Freq |
| Wild type | + | + | + | + | 0.8958 |
| c.2282del4 | Mut | + | + | + | 0.0589 |
| p.R501X | + | Mut | + | + | 0.0334 |
| p.R2447X | + | + | Mut | + | 0.0103 |
| p.S3247X | + | + | + | Mut | 0.0017 |

Haplotype frequencies were calculated using all Central European families. “+” and Mut represent “wild type” and mutant alleles respectively. Freq refers to allelic frequenc<.
