# Supplementary material for: Maternal Filaggrin Mutations Increase the Risk of Atopic Dermatitis in Children: An Effect Independent of Mutation Inheritance
Source: PLoS Genet. 2015 Mar 10;11(3):e1005076. doi: 10.1371/journal.pgen.1005076 (PMC4355615; doi:10.1371/journal.pgen.1005076)
Supplement: S3 Table — (DOCX) [file pgen.1005076.s005.docx]

###### Table S3: Analysis of interaction between child and maternal *FLG* mutations.

|  |  | **Maternal Child Genotype model (MCG)** | | | | | | |
| --- | --- | --- | --- | --- | --- | --- | --- | --- |
| Study |  | R1 (CI) | R2 (CI) | S1 (CI) | γ11 (CI)^a^ |  | *P*_null_^b^ | *P* _vs MCG_^c^ |
| Central Europe |  | 2.57 (2.18-3.04) | 7.97 (5.36-11.87) | 1.55 (1.29-1.87) | - |  | 2.7 x 10^-77^ | - |
| Northern Europe |  | 2.13 (1.67-2.72) | 5.89 (2.67-12.97) | 1.42 (1.11-1.82) | - |  | 1.4 x 10^-17^ | - |
|  |  |  |  |  |  |  |  |  |
|  |  | **MCG-Interaction model** | | | | | | |
| Study |  | R1 (CI) | R2 (CI) | S1 (CI) | γ11 (CI)^a^ |  | *P*_null_^a^ | *P*_Im vs CG_^b^ |
| Central Europe |  | 2.82 (2.26-3.51) | 7.16 (4.64-11.04) | 1.72 (1.34-2.22) | 0.81 (0.56-1.16) |  | 1.6 x 10^-76^ | 0.24 |
| Northern Europe |  | 2.11 (1.54-2.88) | 5.97 (2.61-13.64) | 1.40 (1.00-1.96) | 1.03 (0.63-1.66) |  | 8.1 x 10^-17^ | 0.91 |
|  |  |  |  |  |  |  |  |  |

^a^ γ11 is the interaction term which estimates the disease risk in the child when both mother and child carry one *FLG* mutation. ^b^ *P* value for the comparison of each model versus the null model with no effects. ^c^ *P* value for the comparison of each model versus the Maternal Child Genotype model. CI indicates 95% confidence interval. All results correspond to the combined *FLG* mutations.
