# Supplementary material for: Maternal Filaggrin Mutations Increase the Risk of Atopic Dermatitis in Children: An Effect Independent of Mutation Inheritance
Source: PLoS Genet. 2015 Mar 10;11(3):e1005076. doi: 10.1371/journal.pgen.1005076 (PMC4355615; doi:10.1371/journal.pgen.1005076)
Supplement: S4 Table — (DOCX) [file pgen.1005076.s006.docx]

##### Table S4. Analysis of individual FLG mutations

| **Mutation c.2282del4; Maternal Child Genotype model (MCG)** | | | | | |
| --- | --- | --- | --- | --- | --- |
| Study | R1 (CI) | R2 (CI) | S1 (CI) | *P*_null_^a^ | *P*_MCG vs CG_^b^ |
| Central European | 2.52 (2.06-3.08) | 10.13 (5.41-18.98) | 1.36 (1.08-1.72) | 9.3 x 10^-42^ | 0.009 |
| Northern European | 2.43 (1.79-3.31) | 6.50 (2.24-18.90) | 1.30 (0.94-1.78) | 2.6 x 10^-12^ | 0.12 |
| Meta-analysis  *P*_meta_^c^ | 2.49 (2.11-2.95)  3.2 x 10^-26^ | 9.04 (5.26-15.53)  1.5 x 10^-15^ | 1.34 (1.11-1.62)  0.002 | - | - |
| *P*_het_^d^ | 0.85 | 0.48 | 0.80 |  |  |
|  | | | | | |
| **Mutation p.R501X; Maternal Child Genotype model (MCG)** | | | | | |
| Study | R1 (CI) | R2 (CI) | S1 (CI) | *P*_null_^a^ | *P*_MCG vs CG_^b^ |
| Central European | 2.83 (2.16-3.70) | 3.52 (1.05-11.76) | 1.84 (1.35-2.51) | 5.5 x 10^-30^ | 1.3 x 10^-4^ |
| Northern European | 2.29 (1.47-3.59) | 2934.74 (0-∞)^e^ | 1.09 (0.69-1.72) | 1.9 x 10^-4^ | 0.70 |
| Meta-analysis  *P*_meta_^c^ | 2.67 (2.12-3.37)  7.3 x 10^-17^ | 3.54 (1.06-11.81)  0.04 | 1.56 (1.21-2.02)  6.1 x 10^-4^ | - | - |
| *P*_het_^d^ | 0.43 | 0.79 | 0.06 |  |  |
|  | | | | | |
| **Mutation p.R2447X; Maternal Child Genotype model (MCG)** | | | | | |
| Study | R1 (CI) | R2 (CI) | S1 (CI) | *P*_null_^a^ | *P*_MCG vs CG_^b^ |
| Central European | 2.37 (1.49-3.77) | 8.98 (0.79-102.11) | 1.22 (0.69-2.16) | 2.3 x 10^-5^ | 0.50 |
| Northern European^f^ | 0.60 (0.60-0.60) | 1.21 (1.21-1.21) | 2.23 (2.23-2.23) | 0.09 | 0.01 |

^a^ *P* value for the comparison versus the null model. ^b^ *P* value for the comparison versus the child genotype model. ^c^ *P* value for the meta-analysis of each estimated parameter. ^d^ P value for a test of heterogeneity. ^e^ No homozygous carriers of R501X were found among the Northern European controls leading to a huge estimate of the R2 effect. ^f^ When analyzing the rare *FLG*R2447X mutation in the Northern European population, a warning message was obtained from EMIM indicating that there was insufficient data available to estimate the R1, R2 and S1 parameters. Therefore, no meta-analysis was performed for this mutation. CI indicates the 95% confidence interval.
