# Supplementary material for: Maternal Filaggrin Mutations Increase the Risk of Atopic Dermatitis in Children: An Effect Independent of Mutation Inheritance
Source: PLoS Genet. 2015 Mar 10;11(3):e1005076. doi: 10.1371/journal.pgen.1005076 (PMC4355615; doi:10.1371/journal.pgen.1005076)
Supplement: S5 Table — (DOCX) [file pgen.1005076.s007.docx]

###### Table S5: Frequencies of each of the 4 *FLG* mutations analyzed independently.

|  |  | Central European Population | | | | Northern European population | | | |
| --- | --- | --- | --- | --- | --- | --- | --- | --- | --- |
| c.2282del4 |  | Father | Mother | AD | Control | Father | Mother | AD | Control |
|  | Mutation freq^a^ | 0.0541 | 0.0634 | 0.0830 | 0.0232 | 0.0469 | 0.0508 | 0.0666 | 0.0210 |
|  | +/Mut freq^b^ | 0.1028 | 0.1188 | 0.1358 | 0.0457 | 0.0881 | 0.1017 | 0.1163 | 0.0421 |
|  | Mut/Mut freq^c^ | 0.0027 | 0.0040 | 0.0151 | 0.0003 | 0.0028 | 0 | 0.0084 | 0 |
| p.R501X |  | Father | Mother | AD | Control | Father | Mother | AD | Control |
|  | Mutation freq^a^ | 0.0290 | 0.0384 | 0.0449 | 0.0105 | 0.0171 | 0.0238 | 0.0298 | 0.0111 |
|  | +/Mut freq^b^ | 0.0580 | 0.0768 | 0.0856 | 0.0204 | 0.0286 | 0.0476 | 0.0540 | 0.0221 |
|  | Mut/Mut freq^c^ | 0 | 0 | 0.0021 | 0.0003 | 0.0029 | 0 | 0.0028 | 0 |
| p.R2447X |  | Father | Mother | AD | Control | Father | Mother | AD | Control |
|  | Mutation freq^a^ | 0.0099 | 0.0107 | 0.0127 | 0.0035 | 0.0145 | 0.0145 | 0.0090 | 0.0051 |
|  | +/Mut freq^b^ | 0.0199 | 0.0214 | 0.0236 | 0.0070 | 0.0290 | 0.0289 | 0.0180 | 0.0102 |
|  | Mut/Mut freq^c^ | 0 | 0 | 0.0009 | 0 | 0 | 0 | 0 | 0 |
| p.S3247X |  | Father | Mother | AD | Control | Father | Mother | AD | Control |
|  | Mutation freq^a^ | 0.0013 | 0.0020 | 0.0026 | 0.0009 | 0.0029 | 0.0049 | 0.0043 | na^d^ |
|  | +/Mut freq^b^ | 0.0027 | 0.0040 | 0.0052 | 0.0018 | 0.0059 | 0.0098 | 0.0086 | na^d^ |
|  | Mut/Mut freq^c^ | 0 | 0 | 0 | 0 | 0 | 0 | 0 | na^d^ |

Allele and genotype frequencies of each *FLG*-mutation in fathers and mothers were calculated using all available parents. AD refers to the frequency in the AD-affected children including the families and the unrelated AD-cases (available only in the Central European study). Frequency in controls corresponds to population-based individuals with unknown disease status. ^a^ Refers to the allelic frequency of the mutation. ^b^ Frequency of individuals with heterozygous genotype. ^c^ Frequency of individuals with homozygous or compose heterozygote mutant genotype. ^d^No p.S3247X genotype data was available from the BAMSE controls included in the Northern European population.
