# Supplementary material for: Maternal Filaggrin Mutations Increase the Risk of Atopic Dermatitis in Children: An Effect Independent of Mutation Inheritance
Source: PLoS Genet. 2015 Mar 10;11(3):e1005076. doi: 10.1371/journal.pgen.1005076 (PMC4355615; doi:10.1371/journal.pgen.1005076)
Supplement: S6 Table — (DOCX) [file pgen.1005076.s008.docx]

##### Table S6. Parent-of-origin analysis after exclusion of families with parental history of AD

| **Child genotype model (CG)** | | | | | |
| --- | --- | --- | --- | --- | --- |
| Study | R1 (CI) | R2 (CI) | S1 (CI) | *P*_null_^a^ | *P*_CG vs MG_^b^ |
| Central European | 3.40 (2.89-3.99) | 12.25 (7.73-19.42)) | - | 1.4 x 10^-67^ | - |
| Northern European | 2.61 (1.89-3.62) | 10.78 (3.11-37.31) | - | 2.0 x 10^-09^ | - |
| Meta-analysis  *P*_meta_^c^ | 3.22 (2.79-3.73)  8.8 x 10^-57^ | 12.06 (7.83-18.58)  1.4 x 10^-29^ | - | - | - |
| *P*_het_^d^ | 0.16 | 0.85 |  |  |  |
|  |  | | | | |
| **Maternal Genotype model (MG)** | | | | | |
| Study | R1 (CI) | R2 (CI) | S1 (CI) | *P*_null_^a^ | *P*_CG vs MG_^b^ |
| Central European | 2.88 (2.36-3.52) | 9.61 (5.87-15.74) | 1.42 (1.11-1.81) | 4.4 x 10^-68^ | 0.006 |
| Northern European | 2.34 (1.60-3.42) | 8.92 (2.47-32.24) | 1.29 (0.83-1.99) | 5.8 x 10^-09^ | 0.26 |
| Meta-analysis  *P*_meta_^c^ | 2.76 (2.31-3.29)  3.7 x 10^-29^ | 9.52 (6.01-15.09)  8.2 x 10^-22^ | 1.38 (1.12-1.72)  0.003 | - | - |
| *P*_het_^d^ | 0.34 | 0.92 | 0.71 |  |  |

##### ^a^ P value for the comparison of each model versus the null model with no effects. ^b^ P value for the comparison with the child genotype model. ^c^ P_meta_ refers to the P value for the meta-analysis of each estimated parameter. ^d^ P value for a test of heterogeneity. CI indicates the 95% confidence interval.
