# Supplementary material for: Maternal Filaggrin Mutations Increase the Risk of Atopic Dermatitis in Children: An Effect Independent of Mutation Inheritance
Source: PLoS Genet. 2015 Mar 10;11(3):e1005076. doi: 10.1371/journal.pgen.1005076 (PMC4355615; doi:10.1371/journal.pgen.1005076)
Supplement: S7 Table — (DOCX) [file pgen.1005076.s009.docx]

##### Table S7. Analysis for paternal genotype effects

|  |  | **Children Genotype model (CG)** | | | | | | |
| --- | --- | --- | --- | --- | --- | --- | --- | --- |
| Study |  | R1 (CI) | R2 (CI) | S1 (CI) | Im (CI) |  | *P*_null_^a^ | - |
| Central Europe |  | 3.11 (2.69-3.60) | 10.5 (7.18-15.32) | - | - |  | 4.0 x 10^-74^ | - |
| Northern Europe |  | 2.44 (1.95-3.06) | 7.30 (3.37-15.81) | - | - |  | 9.2 x 10^-17^ | - |
| Meta-analysis  *P*_meta_^c^ |  | 2.90 (2.57-3.27)  1.8 x 10^-65^ | 9.77 (6.96-13.74)  2.4 x 10^-39^ | - | - |  | - | - |
| *P*_het_^d^ |  | 0.08 | 0.41 |  |  |  |  |  |
|  |  |  |  |  |  |  |  |  |
|  |  | **Paternal Child Genotype model (PCG)** | | | | | | |
| Study |  | R1 (CI) | R2 (CI) | S1 (CI) | Im (CI) |  | *P*_null_^a^ | *P*_PCG vs CG_^b^ |
| Central Europe |  | 3.06 (2.60-3.61) | 10.2 (6.88-15.24) | 1.04 (0.86-1.26) | - |  | 5.4 x 10^-73^ | 0.70 |
| Northern Europe |  | 2.36 (1.84-3.02) | 6.84 (3.09-15.14) | 1.10 (0.85-1.42) | - |  | 5.0 x 10^-16^ | 0.48 |
| Meta-analysis  *P*_meta_^c^ |  | 2.83 (2.46-3.24)  3.7 x 10^-49^ | 9.44 (6.62-13.48)  1.2 x 10^-34^ | 1.06 (0.91-1.23)  0.61 | - |  | - | - |
| *P*_het_^d^ |  | 0.08 | 0.37 | 0.74 |  |  |  |  |
|  |  |  |  |  |  |  |  |  |
|  |  | **Imprinting model (Im)** | | | | | | |
| Study |  | R1 (CI) | R2 (CI) | S1 (CI) | Im (CI) |  | *P*_null_^a^ | *P*_Im vs CG_^b^ |
| Central Europe |  | 3.54 (2.95-4.26) | 13.9 (8.75-21.94) | - | 0.76 (0.58-0.98) |  | 6.4 x 10^-74^ | 0.002 |
| Northern Europe |  | 2.70 (2.05-3.55) | 9.04 (3.85-21.22) | - | 0.81 (0.57-1.16) |  | 3.3 x 10^-16^ | 0.25 |
| Meta-analysis  *P*_meta_^c^ |  | 3.26 (2.80-3.79)  1.2 x 10^-51^ | 12.58 (8.40-18.86)  2.9 x 10^-34^ | - | 0.78 (0.63-0.96)  0.009 |  | - | - |
| *P*_het_^d^ |  | 0.11 | 0.39 |  | 0.75 |  |  |  |

^a^ *P* value for the comparison of each model versus the null model with no effects. ^b^ *P* value for the comparison of each model versus the Child Genotype model. ^c^ *P* value for the meta-analysis of each estimated parameter (see Supplementary methods). ^d^ P value for a test of heterogeneity. CI indicates 95% confidence interval. All results correspond to the combined *FLG* mutations. This analysis was performed after exchanging the maternal and paternal genotypes. Thus, S1 and the Im represent the effect of the paternal genotype and of mutation inheritance from the father, respectively.
