# Supplementary material for: Maternal Filaggrin Mutations Increase the Risk of Atopic Dermatitis in Children: An Effect Independent of Mutation Inheritance
Source: PLoS Genet. 2015 Mar 10;11(3):e1005076. doi: 10.1371/journal.pgen.1005076 (PMC4355615; doi:10.1371/journal.pgen.1005076)
Supplement: S8 Table — (DOC) [file pgen.1005076.s010.doc]

##### Table S8. Parent-of-origin analysis with one single AD-affected child per family

| **Child genotype model (CG)** | | | | | |
| --- | --- | --- | --- | --- | --- |
| Study | R1 (CI) | R2 (CI) | S1 (CI) | *P*_null_^a^ | *P*_MCG vs CG_^b^ |
| Central European | 3.42 (2.92-4.00) | 11.71 (7.48-18.34) | - | 1.8 x 10^-69^ | - |
| Northern European | 2.64 (2.01-3.47) | 10.84 (4.35-26.94) | - | 8.1 x 10^-15^ | - |
| Meta-analysis  *P*_meta_^c^ | 3.20 (2.79-3.67)  4.2 x 10^-62^ | 11.53 (7.71-17.25)  1.1 x 10^-32^ | - | - | - |
| *P*_het_^d^ | 0.11 | 0.88 |  |  |  |
|  |  |  |  |  |  |
| **Maternal Child Genotype model (MCG)** | | | | | |
| Study | R1 (CI) | R2 (CI) | S1 (CI) | *P*_null_^a^ | *P*_MCG vs CG_^b^ |
| Central European | 2.82 (2.32-3.42) | 8.80 (5.45-14.20) | 1.50 (1.19-1.90) | 7.6 x 10^-71^ | 6.4 x 10^-4^ |
| Northern European | 2.30 (1.69-3.14) | 8.72 (3.39-22.40) | 1.36 (0.98-1.87) | 1.0 x 10-^14^ | 0.066 |
| Meta-analysis  *P*_meta_^c^ | 2.66 (2.26-3.14)  2.7 x 10^-31^ | 8.78 (5.73-13.46)  2.1 x 10^-23^ | 1.45 (1.20-1.75)  1.1 x 10^-4^ | - | - |
| *P*_het_^d^ | 0.28 | 0.99 | 0.61 |  |  |

^a^ *P* value for the comparison of each model versus the null model with no effects. ^b^ *P* value for the comparison of each model versus the child genotype model. ^c^ *P* value for the meta-analysis of each estimated parameter. ^d^ P value for a test of heterogeneity. CI indicates the 95% confidence interval.
