# Supplementary material for: Maternal Filaggrin Mutations Increase the Risk of Atopic Dermatitis in Children: An Effect Independent of Mutation Inheritance
Source: PLoS Genet. 2015 Mar 10;11(3):e1005076. doi: 10.1371/journal.pgen.1005076 (PMC4355615; doi:10.1371/journal.pgen.1005076)
Supplement: S9 Table — (DOCX) [file pgen.1005076.s011.docx]

|  | Parameters included | | | |
| --- | --- | --- | --- | --- |
| Model | R1 | R2 | Im | S1 |
| Null | - | - | - | - |
| Child Genotype (CG) | + | + | - | - |
| Maternal Child Genotype (MCG) (MCG) (MCG) | + | + | - | + |
| Imprinting (Im) | + | + | + | - |
| Full | + | + | + | + |

**Table S9. Step-by-step analysis with PREMIM/EMIM**

R1, R2, Im and S1 are the risk parameters that were successively included in the PREMIM/EMIM models. “-“ and “+” indicate which parameters were excluded or included on each model, respectively.
