# Supplementary material for: Maternal Filaggrin Mutations Increase the Risk of Atopic Dermatitis in Children: An Effect Independent of Mutation Inheritance
Source: PLoS Genet. 2015 Mar 10;11(3):e1005076. doi: 10.1371/journal.pgen.1005076 (PMC4355615; doi:10.1371/journal.pgen.1005076)
Supplement: S10 Table — (DOCX) [file pgen.1005076.s012.docx]

###### Table S10. Models testing interaction between the child and the maternal genotypes

|  | Parameters included | | | |
| --- | --- | --- | --- | --- |
| Model | R1 | R2 | S1 | γ11 |
| Maternal Child Genotype (MCG) (MCG) (MCG) | + | + | + | - |
| MCG-Interaction | + | + | + | + |

R1, R2, S1 and γ11 are the risk parameters that were excluded “-“ or included “+” on each model.
